# Determinants of community pharmacists’ information gathering and counseling practices during the management of minor ailments

**DOI:** 10.1016/j.jsps.2021.07.016

**Published:** 2021-07-21

**Authors:** Ahmed Mohamed Makhlouf, Mohamed Izham Mohamed Ibrahim, Ahmed Awaisu, Saseendran Kattezhathu Vyas, Kazeem Babatunde Yusuff

**Affiliations:** a Department of Clinical Pharmacy and Practice, College of Pharmacy, QU Health, Qatar University, Doha, Qatar; b Wellcare Group, Airport Road, Doha, Qatar

**Keywords:** Minor ailments, Community pharmacists, Information gathering, Counseling, Determinants

## Abstract

**Objective:**

To assess the determinants of community pharmacists’ information gathering and counseling practices during the management of minor ailments in Qatar.

**Method:**

A cross-sectional study of 305 community pharmacists was conducted with a pre-tested 27-item questionnaire. Bivariate logistic regression was used to identify the determinants of information gathering and counseling practices.

**Results:**

The response rate was 92.5% (282/305). A majority of the respondents (68.1%) were males, within the age range of 31–40 years (55.3%), work for chains pharmacies (77.3%), and were predominantly of foreign nationalities (94.7%). Patients’ identity (91.1%), age (92.2%), symptoms (92.6%) and duration of symptoms (89.3%) were most frequent information gathered, while dose (99%), frequency (97.8%), route of administration (95.7%), and duration of use (92.9%) were the most frequent counseling information. Median information gathering score was significantly higher in females and among community pharmacists in chain pharmacies (p < 0.05), while median counseling practice scores were significantly higher among in chain pharmacies (p < 0.05). Consultation time of 6–10 min (OR = 1.75, 95% CI: 1.02–3.0, p = 0.04) and female gender (OR = 2.10, 95% CI: 1.16–3.79, p = 0.01) were significant determinants of information gathering, while age group (31–40 years) (OR = 1.84, 95% CI: 1.05–3.22, p = 0.03) and consultation time (6–10 min) (OR = 2.24, 95% CI: 1.31–3.86, p = 0.003) were significant determinants of counseling practices.

**Conclusion:**

The significant determinants of community pharmacists’ Information gathering and counseling practices during the management of minor ailments were female gender and consultation time (6–10 min), and age group (31–40 years) and consultation time (6–10 min) respectively.

## 1 Introduction

Community pharmacists are trusted healthcare professionals with adequate competency to manage the effective and safe use of medications at the primary care level. They are uniquely positioned to manage medical conditions that are perceived as minor, uncomplicated and can be handled with guided self-medication or self-care (Morgan, 2017, You et al., 2011). This is due to their geographic availability and neighborhood presence, the ease of social access and their perception by patients as trusted and ethical healthcare professional (Coelho and Costa, 2014, World Health Organization (WHO), 1988). The combination of these factors provide an excellent opportunity for timely access to functional care and appropriate counseling that may enhance effective and safe management of minor ailments (World Health Organization and Office, 2009, Hughes, 2004, Royal Pharmaceutical Society of Great Britain (RPSGB), 2018). Several studies have reported the positive impact of community pharmacists’ involvement in the management of minor ailments, and these include high rates of symptom resolution, lower treatment costs, adequate patient satisfaction with service delivery, reduction of patient delays and more efficient use of healthcare resources (Watson et al., 2014, Watson et al., 2015, Paudyal et al., 2013, Porteous et al., 2006).

However, the effective management of minor ailments by community pharmacists is predicated on adequate information gathering and comprehensive provision of information to patients during counseling (Okumura et al., 2014, van Eikenhorst et al., 2016). Indeed, inadequate retrieval or lack of relevant patient information that will enable community pharmacists to correctly assess and diagnose minor ailments may compromise the quality of care provided or lead to recommendation of treatments that are inappropriate, unnecessary or potentially harmful (Eickhoff et al., 2012). In addition, the inadequate provision of counseling information that should ensure appropriate use of recommended medications may compromise service quality and potentially expose patients to harm (Kroeger, 2001). For, Instance, a study done in Massachusetts, United States showed that inappropriate use of non-prescription medications by consumers accounted for 170,000 hospitalizations per year with an estimated annual costs of USD 750 million; and at least half of avoidable clinical and financial burden could have been prevented with counseling and patient education (Bosse et al., 2012). In addition, community pharmacists are obligated based on the pharmaceutical care / therapeutic planning competency acquired during the undergraduate pharmacy training and practice benchmarks provided by national and international organizations to obtain critical patient information during clinical encounters. These include patients’ identity, age, symptoms, medical and medication history and allergy history to enable adequate assessment and therapeutic planning including those related to minor ailments (Pharmaceutical Services Division (PSD), Ministry of Health, Malaysia, 2016, International Pharmaceutical Federation (FIP), 1998, World Health Organization (WHO), 1996). Furthermore, community pharmacists are expected to provide a detailed educational and counseling information to enable appropriate medication use and these include dose, regimen, duration, storage conditions, potential side effects and drug interactions, and probable contraindications (Pharmaceutical Society of Australia, 2018).

Several studies have documented the inconsistent and inadequate information gathering and counseling practices by community pharmacists during minor ailments-related encounters (van Eikenhorst et al., 2016, Kroeger et al., 2001, Brata et al., 2015a). Two systematic reviews of the information gathering process and provision of appropriate advice during self-medication encounters in community pharmacies in developing countries concluded that the rates of information gathering and the types of information gathered were inconsistent and generally inadequate (Brata et al., 2013, Brata et al., 2015b). In addition, the provision of inappropriate advice was reported by the majority of the studies included in the reviews. Furthermore, patients’ identity and symptoms were the most frequently documented, while medical and medication history were the least documented (Brata et al., 2013). However, literature search did not reveal any study focused on the determinants of information gathering and counseling practices of community pharmacists during the management of minor ailments. In fact, only one recent study conducted in community pharmacies in Germany reported lack of patient’s interest, time constraint and language barrier as the major factors mentioned as responsible for poor counseling practices related to the management of self-medication with non-prescription medicines (Seiberth et al., 2020). In addition, Cassie et al., in a qualitative assessment of barriers to information gathering by community pharmacists and medicines counter assistants in Scotland identified factors such as lack of privacy, belief about patient safety, inadequate communication skills, perception of pharmacists’ role and behavioral regulatory factors such as the lack or non-usage of standard operating procedure, organizational culture and training (Cassie et al., 2019). Hence, findings of the current study may provide new perspectives that will add significantly to knowledge in the research area. In addition, the study may also provide data that will be useful in designing future intervention studies related to improving community pharmacists’ information gathering and counseling practices during the management of minor ailments. Therefore, the objectives of the study were to assess community pharmacists’ self-reported information gathering and counseling practices during the management of minor ailments, and to identify the significant determinants of these practices in Qatar.

## 2 Methods

### 2.1 Study design

A questionnaire-based cross-sectional assessment of the information gathering and counseling practices of community pharmacists during the management of minor ailments in Qatar was conducted between 16 June 2019 and 28 August 2019. Qatar is a Middle Eastern country with an estimated population of 2.7 million and consists of eight municipalities (United Nations, 2020).

### 2.2 Study population, sampling and sample size

All the licensed community pharmacists whose names were present on the list obtained from the Ministry of Public Health in Qatar (n = 1016) constituted the sampling frame and were purposively targeted for the study. The community pharmacists working in chains constituted 75% of the names on the list, while those working in independent community pharmacies accounted for the balance (25%). To be eligible for inclusion, licensed community pharmacists must be in practice for at least a year in either independent or chain pharmacies in Qatar, and able to speak and write in English language. The *a priori* calculation of the required sample of community pharmacists was done with Raosoft® Online Calculator, and the parameters used include the number of the community pharmacists licensed in Qatar (n = 1016), alpha level (5%), confidence level (95%) and a response distribution of 50%. This gave a required sample size of 279 community pharmacists but 10% was added to account for possible decline to participate and this resulted in a total sample size of 305 community pharmacists. The final purposive sampling of community pharmacists was based on a 75:25 ratio (chain vs. independent pharmacies).

### 2.3 Tools development and validation procedure

The information gathering and counseling practices of community pharmacists were assessed with a 27-item questionnaire that was developed after a thorough review of relevant published literature (van Eikenhorst et al., 2016, Kroeger et al., 2001, Brata et al., 2015a, Brata et al., 2015b). The questionnaire consisted of three sections including demographics and community pharmacists’ workload related to minor ailments (A), information gathering related to minor ailments (B), and counseling activities during minor ailment encounters (C). The demographics and workload data include gender, age group, nationality, community pharmacy type, years of experience, highest pharmacy degree, average consultation time for minor ailments, average number of customers per daily shift, number of customers with minor ailments per daily shift, and follow-up planning and documentation of activities related minor ailments. The information gathering data collected include age and identity of customers, symptoms and duration of minor ailments, medical and medication history allergy history. The data gathered on counseling information include dose, frequency, duration and route of administration of medicines recommended for minor ailments, storage conditions, side effects, contraindications and drug interactions, and referral to a physician when necessary.

The study participants were asked to rank their responses to the items in Section B and C on a 5-point Likert-type scale (always = 5, very often = 4, sometimes = 3, rarely = 2 and never = 1). The minimum and maximum obtainable scores for sections B (9 items) and C (8 items) were 45 and 40 respectively. In addition, the scores obtained for the information gathering and counseling practice scores were categorized into two including ≥ median and below < median. Three experienced faculty members of the College of Pharmacy, Qatar University and a senior manager in one of the community pharmacy chains in Qatar assessed the content validity of the questionnaire. In addition, the questionnaire was pre-tested with a convenient sample of eight community pharmacists for structure, clarity, completeness and time for completion. The pretest data were not included in the final results. The reliability analysis of the sections B and C of the final questionnaire showed Cronbach’s alpha coefficient of 0.89 and 0.83, respectively.

### 2.4 Data collection procedure

Data collection was preceded by initial visits with introductory letters to the pharmacy managers of the independent and chain pharmacies to maximize cooperation and participation of the sampled community pharmacists. The introductory letters provided key details such as the title, purpose and the potential impact of the study. In addition, a short video about the management of minor ailments by community pharmacists in the United Kingdom (UK) was provided to stimulate interest and enhance participation. The study participants were approached at their premises and signed informed consent forms indicating voluntary participation were obtained before the questionnaire were provided. In addition, the participants were assured that their responses would be kept confidential and used only for research purposes. Completed questionnaires were collected on the same day, while reminders were sent via phone calls and/or text messages to the participants who did not complete the questionnaires at the first visit. Clarifications on any of the questionnaire items were provided by data collectors as necessary. The Qatar University’s Institutional Review Board approved the study protocol before the commencement of data collection (QU-IRB reference number 1074-E/19, dated 03 May 2019).

### 2.5 Data analysis

Data analysis was done with the SPSS version 26.0. for Windows (IBM SPSS Statistics for Windows, 2019, Version 26.0. Armonk, NY: IBM Corp.). The data normality was assessed with the Shapiro-Wilk test and descriptive parameters such as frequencies, percentages, mean ± SD or median (IQR) were used as appropriate. Statistical analysis of information gathering and counseling practices across demographics and workload groups was done with Man-Whitney U and Kruskal Wallis tests. Binary logistic regression was used to identify the determinants of community pharmacists’ information gathering and counseling practices. The level of statistical significance was set at p ≤ 0.05.

## 3 Results

Two hundred and eighty-two of the 305 community pharmacists sampled completed the questionnaires (response rate, 92.5%). The demographic characteristics of the community pharmacists sampled are as shown in Table 1. The result of Shapiro Wilk test for the information gathering and counseling practices score showed non-normal distribution (0.87, p < 0.001) and this was taken into consideration in the choice of descriptive and inferential statistical methods for analysis. A majority of the community pharmacists were males (68.1%; 192/282), within the age range of 31–40 years (55.3%; 156/282), work for chains pharmacies (77.3%; 218/282) and consisted mainly of 4 nationalities (94.7%; 267/282) including Indian (43.3%), Egyptian (35.1%), Sudanese (8.9%) and Filipino (7.4%). The most frequent highest pharmacy degree was BSc/BPharm (81.6%; 230/282) and the median (IQR) for the years of experience was 7 (4–10).

**Table 1:** Demographics and community pharmacists’ perceived workload related to minor ailments in chains and independent community pharmacies in Qatar (N = 282).

**Item**	**n (%)**
**Gender**	
Male	192 (68.1)
Female	90 (31.9)
**Age group (years)**	
21–30	101 (35.8)
31–40	156 (55.3)
41–50	18 (6.4)
51–60	5 (1,8)
>60	2 (0.7)
**Nationality**	
Indian	122 (43.2)
Egyptian	99 (35.1)
Sudanese	25 (8.9)
Filipino	21 (7.4)
Jordanian	5 (1.8)
Syrian	4 (1.4)
Pakistani	4 (1.4)
Palestinian	1 (0.4)
Canadian	1 (0.4)
**Experience (years), Median (IQR)**	7 (4, 10.3)
**Highest Pharmacy Degree**	
BSc/BPharm	230 (81.6)
MSc Pharm	29 (10.3)
PharmD	10 (3.5)
Diploma	13 (4.6)
**Type of Community Pharmacy**	
Independent	64 (22.7)
Chains	218 (77.3)
**# of Customers per daily shift**	
1–10 Customers	3 (1.1)
11–20 Customers	21 (7.4)
21–30 Customers	57 (20.2)
>30 Customers	201 (71.3)
**# of customers with minor ailments per daily shift**	
1–10 Customers	69 (24.5)
11–20 Customers	80 (28.4)
21–30 Customers	74 (26.2)
>30 Customers	59 (20.9)
**Consultation time for minor ailments**	
<=5 min	113 (40.1)
6–10 min	146 (51.8)
11–15 min	19 (6.7)
16–20 min	4 (1.4)
**Documentation of minor ailment services**	
Yes	129 (45.7%)
No	153 (54.3)

Community pharmacists’ perceived workload related to minor ailments and information gathering practices are shown in Table 1. The most frequent estimate of the number of customers seen per daily shift (30 or more) and the number that presents with minor ailments among these customers (11–30) were 71.3% (201/282) and 54.6% (154/282) respectively. The most frequent estimate of consultation time for minor ailment was 6–10 min (51.8%, 146/282), followed by <5 min (40.1%, 113/282). About 40% (111/282) of the community pharmacists reported that they always or often follow-up with patients on the outcomes of the recommendation made to resolve minor ailments, and about 46% (129/282) claimed that they document some of the activities related to their management of minor ailments. In addition, about 67% (190/282) of the respondents reported that they sometimes make referral of minor ailments to physicians when required, while only about 25% (71/282) always or often do so.

The median (IQR) score for information gathering practices related to minor ailments was 33 (29–35). Descriptive analysis of the community pharmacists’ responses showed that they always or often ask for the following during the management of minor ailments: patient identity (91.1%), age (92.2%), symptoms (92.6%), duration of symptoms (89.3%), other medical conditions (79.1%), past or current medications (83.1%) and possible allergies (75.2%) (Table 2).

**Table 2:** Community pharmacists’ perceived information gathering and counseling practices during the management of minor ailment in Qatar [N = 282].

**Item**	**Never, n (%)**	**Rarely, n (%)**	**Sometimes, n (%)**	**Very Often, n (%)**	**Always, n (%)**
**Information gathering practices**					
Customer’s identity	0 (0)	3 (1.1)	21 (7.4)	43 (15.2)	214 (75.9)
Customer’s age	0 (0)	4 (1.4)	17 (6.0)	56 (19.9)	204 (72.3)
Symptoms	0 (0)	5 (1.8)	14 (5.0)	40 (14.2)	222 (78.7)
Duration of symptoms	1 (0.4)	5 (1.8)	23 (8.2)	54 (19.1)	198 (70.2)
Medical history	0 (0)	7 (2.5)	52 (18.4)	77 (27.3)	146 (51.8)
Medication history	0 (0)	9 (3.2)	36 (12.8)	56 (19.9)	180 (63.8)
Allergy history	1 (0.4)	22 (7.8)	47 (16.7)	76 (27.0)	136 (48.2)
Previous follow-up	4 (1.4)	20 (7.1)	144 (51.1)	52 (22.0)	49 (17.4)
Previous referral	0 (0)	18 (6.4)	190 (67.4)	56 (19.9)	15 (5.3)
**Counseling practices**					
Drug dose	0 (0)	1 (0)	2 (0.7)	36 (12.8)	243 (86.2)
Frequency of administration	0 (0)	0 (0)	6 (2.1)	32 (11.3)	244 (86.5)
Route of administration	0 (0)	1 (0.4)	11 (3.9)	39 (13.8)	231 (81.9)
Duration of treatment	0 (0)	1 (0.4)	18 (6.4)	49 (17.4)	214 (75.9)
Storage condition	1 (0.4)	9 (3.2)	64 (22.7)	86 (30.5)	122 (43.3)
Possible side effects	1 (0.4)	15 (5.3)	86 (30.5)	94 (33.3)	86 (30.5)
Possible contraindication	0 (0)	25 (8.9)	93 (33.0)	78 (27.7)	85 (30.1)
Possible drug interactions	0 (0)	21 (7.4)	100 (35.5)	78(27.7)	83 (29.4)

The counseling information provided by community pharmacists during the management of minor ailments are shown in Table 2. The median (IQR) score for community pharmacists’ counseling practices was 35 (32–38). Community pharmacists reported that information regarding the following were always or often provided: dose (99%), frequency 97.8%), route of administration (95.7%), and duration of use (92.9%). Others include contraindication (57.8%) and potential drug interactions (57.1%).

The median information gathering score was significantly higher in female community pharmacists relative to males (33) (34 vs. 33; *U* = 6828, *p* = 0.004), but there was no significant difference in median counseling practice score between male and female community pharmacists (35 vs 35; *U* = 8497, *p* = 0.822). Furthermore, the median information gathering and counseling practice scores were significantly higher among community pharmacists working for chain pharmacies relative to independent (33 vs. 30; *U* = 4260, *p* = 0.0001) and (36 vs. 33; *U* = 5432.5, *p* = 0.007) respectively. In addition, the median information gathering score was significantly higher among community pharmacists with BSc/BPharm (33) and MSc (32) relative to those with Diploma (28) and PharmD (28) (*H* = 14.3, *p* = 0.003), but there was no significant difference in median counseling practice score (BSc/BPharm: 36, MSc: 34, PharmD: 32, Diploma: 32) (*H* = 6.87, *p* = 0.08). Furthermore, the median counseling score was significantly different across age groups (21–30 years: 34; 31–40 years: 36; 41–50 years: 32; 51–60 years: 32) (*H* = 12.9, *p* = 0.01), but not median information gathering scores (21–30 years: 33; 31–40 years: 33; 41–50 years: 32.5; 51–60 years: 33) (*H* = 2.24, *p* = 0.69). There were no clear trends in the strength and direction of association as shown by the result of the coefficients (B) in Table 3, and binary logistic regression analysis showed that minor ailment consultation time of 6–10 min (OR = 1.75, 95% CI: 1.02–3.0, p = 0.04) and female gender (OR = 2.10, 95% CI: 1.16–3.79, p = 0.01) were significant determinants of information gathering practice (p < 0.05) (Table 3). On the other hand, age group (31–40 years) (OR = 1.84, 95% CI: 1.05–3.22, p = 0.03) and consultation time (6–10 min) (OR = 2.24, 95% CI: 1.31–3.86, p = 0.003) were the significant determinants of community pharmacists’ counseling practices during the management of minor ailments (Table 4).

**Table 3:** Binary logistic regression of the determinants of community pharmacists’ information gathering practices during the management of minor ailments in Qatar [N = 282]

		Information gathering						95% CI for Exp(B)		
Item	Categories (n)	<Median (<33)n (%)	≥Median (≥33) n (%)	B	SE	Wald	Exp(B)	Lower	Upper	P-value
**Gender**	Male (192)	89 (46.4)	103 (53.6)				1(reference)			
	Female (90)	30 (33.3)	60 (66.7)	0.739	0.302	5.995	2.095	1.159	3.786	0.014*
**Age groups (years)**	21–30 (101)	47 (46.5)	54 (53.5)				1(reference)			
	31–40 (156)	60 (38.5)	96 (61.5)	0.477	0.290	2.694	1.611	0.912	2.846	0.101
	41–50 (18)	9 (50)	9 (50)	−0.295	0.563	0.274	0.745	0.247	2.247	0.601
	51–60 (5)	2 (40)	3 (60)	0.235	1.030	0.052	1.265	0.168	9.516	0.820
	>60 (2)	1 (50)	1 (50)	0.296	1.494	0.039	1.344	0.072	25.135	0.843
**Highest pharmacy degree**	Diploma (13)	7 (53.8)	6 (46.2)				1(reference)			
	BSc/BPharm (230)	87 (37.8)	143 (62.2)	0.739	0.604	1.497	2.094	0.641	6.839	0.221
	Pharm D (10)	10 (100)	0 (0)	−20.815	12208.941	0.000	0.000	0.000		0.999
	MSc Pharm (29)	15 (51.7)	14 (48.3)	0.094	0.711	0.017	1.098	0.273	4.424	0.895
**No. of customers per daily shift**	1–10 (3)	1 (33.3)	2 (66.7)				1(reference)			
	11–20 (21)	12 (57.1)	9 (42.9)	−1.141	1.391	0.673	0.319	0.021	4.881	0.412
	21–30 (57)	23 (40.4)	34 59.6)	−0.425	1.351	0.099	0.654	0.046	9.231	0.753
	>30 (201)	83 (41.3)	118 (58.7)	−0.211	1.340	0.025	0.810	0.059	11.199	0.875
**No. of customers with MAs per daily shift**	1–10 (69)	30 (43.5)	39 (56.5)				1(reference)			
	11–20 (80)	31 (38.7)	49 (61.3)	0.395	0.382	1.070	1.485	0.702	3.139	0.301
	21–30 (74)	38 (51.4)	36 (48.6)	−0.372	0.411	0.822	0.689	0.308	1.542	0.365
	>30 (59)	20 (33.9)	39 (66.1)	0.059	0.441	0.018	1.060	0.447	2.515	0.894
**Consultation time for MAs (minutes)**	<=5 (113)	59 (52.2)	54 (47.8)				1(reference)			
	6–10 (146)	49 (33.6)	97 (66.4)	0.557	0.276	4.054	1.745	1.015	3.000	0.044*
	11–15 (19)	10 (52.6)	9 (47.4)	−0.030	0.565	0.003	0.971	0.321	2.938	0.958
	16–20 (4)	1 (25)	3 (75)	0.763	1.207	0.399	2.144	0.201	22.858	0.528

NB: 33 (median score) is the cutoff point for information gathering practices (maximum value is 35); MAs = Minor ailments B = Coefficient; SE = Standard Error; Exp(B) = Exponentiation of coefficient; CI = Confidence Interval; * p < 0.05 (statistically significant).

**Table 4:** Binary logistic regression of the determinants of community pharmacists’ counseling practices during the management of minor ailment in Qatar [N = 282].

		Counseling practice						95% CI for Exp(B)		
Item	Categories (n)	<median (<35) n (%)	≥median (≥35) n (%)	B	SE	Wald	Exp(B)	Lower	Upper	P-value
**Gender**	Male (192)	87 (45.3)	105 (54.7)				1(reference)			
	Female (90)	44 (48.9)	46 (51.1)	−0.149	0.289	0.266	0.862	0.490	1.517	0.606
**Age groups (years)**	21–30 (101)	54 (53.5)	47 (46.5)				1(reference)			
	31–40 (156)	63 (40.4)	93 (59.6)	0.609	0.285	4.573	1.839	1.052	3.216	0.032*
	41–50 (18)	12 (66.7)	6 (33.3)	−0.685	0.574	1.424	0.504	0.164	1.553	0.233
	51–60 (5)	1 (20)	4 (80)	2.374	1.456	2.658	10.739	0.619	186.403	0.103
	>60 (2)	1 (50)	1 (50)	0.024	1.655	0.000	1.024	0.040	26.232	0.989
**Highest pharmacy degree**	Diploma (13)	6 (46.2)	7 (53.8)				1(reference)			
	BSc/BPharm (230)	101 (43.9)	129 (56.1)	0.046	0.612	0.006	1.047	0.315	3.477	0.940
	Pharm D (10)	8 (80)	2 (20)	−1.043	1.012	1.064	0.352	0.048	2.559	0.302
	MSc Pharm (29)	16 (55.2)	13 (44.8)	−0.810	0.734	1.219	0.445	0.106	1.874	0.270
**No. of customers per daily shift**	1–10 (3)	1 (33.3)	2 (66.7)				1(reference)			
	11–20 (21)	12 (57.1)	9 (42.9)	−1.162	1.382	0.706	0.313	0.021	4.699	0.401
	21–30 (57)	21 (36.8)	36 63.2)	−0.207	1.346	0.024	0.813	0.058	11.373	0.878
	>30 (201)	97 (48.3)	104 (51.7)	−0.943	1.336	0.498	0.390	0.028	5.346	0.481
**No. of customers with MAs per daily shift**	1–10 (69)	32 (46.4)	37 (53.6)				1(reference)			
	11–20 (80)	40 (50)	40 (50)	0.133	0.378	0.123	1.142	0.544	2.395	0.725
	21–30 (74)	32 (43.2)	42 (56.8)	0.682	0.421	2.628	1.978	0.867	4.515	0.105
	>30 (59)	27 (45.8)	32 (54.2)	0.189	0.438	0.186	1.208	0.512	2.849	0.666
**Consultation time (minutes)**	≤5 (113)	66 (58.4)	47 (41.6)				1(reference)			
	6–10 (146)	53 (36.3)	93 63.7)	0.808	0.276	8.554	2.244	1.305	3.856	0.003*
	11–15 (19)	9 (47.4)	10 (52.6)	0.146	0.558	0.068	1.157	0.388	3.452	0.794
	16–20 (4)	3 (75)	1 (25)	−1.784	1.326	1.811	0.168	0.012	2.258	0.178

NB: 35 (median score) is the cutoff point for counseling practices (maximum is 40); MAs = Minor ailments B = Coefficient; SE = Standard Error; Exp (B) = Exponentiation of coefficient; CI = Confidence Interval; * p < 0.05 (statistically significant).

## 4 Discussion

Female gender was identified as a significant determinant of information gathering as female community pharmacists had twice the odds of obtaining higher information gathering score relative to males. In addition, Information gathering practices was significantly better among female community pharmacists, and this is despite that they constituted only about a third of respondents. This finding is significant and, to our knowledge, being reported for the first time. It is not readily clear what factors may have contributed to this observation, but it has been reported that female community pharmacists seem to have better communication and interpersonal skills, and hence able to better establish good rapport with patients and gain trust, and this may have contributed to the better information gathering observed among females (Pelicano-Romano et al., 2013). However, no significant difference was observed in counseling practice between males and females, and this may have been due to the impact of system- and job behavior-related deficits that could potentially affect community pharmacists’ motivation to counsel in a consistently effective manner (Tully et al., 2011, Svarstad et al., 2004, Foroughinia and Zarei, 2016).

The significantly higher information gathering score observed among community pharmacists working in chain pharmacies is unsurprising and may be due to organizational practice and culture. This is because organizational policies and culture significantly influences employee work-related behavior (Tsai, 2011). For instance, Cassie et al., in a qualitative study of the determinants of information gathering to guide the management of Over-the-counter consultations in community pharmacies identified behavioral regulation associated with organizational policies such as the use of a standard operating procedure as a key factor that shaped information gathering practices. In addition, better access to training for community pharmacists in organizational settings such as in chain pharmacies was also probably contributory (Cassie et al., 2019).

Consultation time was identified as a significant determinant of information gathering as community pharmacists who spent 6 to 10 min had twice the odds of reporting higher information gathering scores. This is probably due to the sufficiency of time afforded community pharmacists to build rapport, gain trust and gather relevant patient information during the management of minor ailments (Foroughinia and Zarei, 2016). Furthermore, the significantly better counseling practices observed among community pharmacists working for chain pharmacies may also be related to organizational policies and practices rather than individual preferences (Cassie et al., 2019).

Similarly, consultation time and community pharmacists’ age group were reported as significant determinants of counseling practices as community pharmacists who reported duration of 6–10 min for minor ailments consultation and those in the 31–40 years age group had twice the odds of reporting higher scores for counseling practice. These findings underscore the importance of sufficiency of time for minor ailments-related consultations by community pharmacists to ensure comprehensive counseling that prevents inappropriate medication use. In addition, the observed impact of age group is unsurprising as community pharmacists who are relatively younger are probably recent graduates who are more likely to be exposed to patient-oriented pharmacy curriculum that is focused on developing therapeutic communication skills, and hence may have more self-awareness to counsel patients comprehensively during the management of minor ailments (Pelicano-Romano et al., 2013).

Overall, the findings of the current study suggest that the frequency of information gathering and counseling by community pharmacists during the management of minor ailments seem adequate for certain components, but inadequate for others. Hence, significant room still exist for improvement to ensure consistency of service delivery. These findings are consistent with those of previous studies in similar settings that reported that community pharmacists’ information gathering related to minor ailments were incomplete and inconsistently done, and may negatively affect the quality of recommendations made to customers (van Eikenhorst et al., 2016, Brata et al., 2015a, Brata et al., 2013). For instance, the frequency of information gathered appeared consistently higher for patients’ age and identity, signs, symptoms, and duration of therapy. On the contrary, gathering of past medical and medication history and allergy history were relatively less in frequency, and this may potentially expose patients to adverse events associated with drug-drug and/or drug-disease interactions. Similarly, the trend observed in the types of counseling information provided by community pharmacists is also consistent with published studies conducted in similar settings (Kroeger et al., 2001, Schneider et al., 2009, Netere et al., 2018, Puspitasari et al., 2009). The observed inadequacy in the frequency of counseling information could potentially compromise patient safety and increase the risks of medication use-related harms (Eickhoff et al., 2012, Bosse et al., 2012). Therefore, there is a significant room for improvement in these critical areas as adequate information gathering and patient counseling remain crucial responsibilities of community pharmacists that will enhance effective and safe recommendations and improve overall patient outcomes.

The inconsistencies in community pharmacists’ information gathering and counseling practices may be related to probable lack or non-usage of a standard protocol that specifically guides and/or serves as a reminder to community pharmacists during the management of minor ailments (Royal Pharmaceutical Society of Great Britain (RPSGB), 2018, Watson et al., 2014). This possibility also appeared supported by the fact that only about 40% of the community pharmacists reported following up with patients on the outcomes of prior recommendations made, and only about half reported the documentation of activities related to management of minor ailments. Perhaps, the availability and use of a minor ailment-specific standard protocol may improve community pharmacists’ information gathering and counseling practices. The international benchmarks on good pharmacy practice developed by the World Health Organization (WHO) and the International Pharmaceutical Federation (FIP) could be used as a template for the development of such specific protocols International Pharmaceutical Federation (FIP), 1998, World Health Organization (WHO), 1996).

The study findings should be interpreted in the light of the limitations including the use of non-probability sampling method. However, the sampling distribution of the study participants was authentic and mirrored the actual proportional representation of community pharmacists in Qatar. Furthermore, social desirability and self-report bias may have influenced the community pharmacists’ response as this was a questionnaire-based survey. However, the high internal consistency of the data collection tool and the striking similarity of the trends in the study findings with those reported by previous studies is noteworthy, and probably suggest that the findings presented are valid and reliable. In addition, social desirability bias appeared not to have affected the relatively lower frequencies reported by community pharmacists for allergy history, medical and medication history (information gathering practices), and storage condition, side effects, drug interactions and contraindication (counseling practices). Finally, the study was focused mainly on community pharmacists and thus all the factors included in the regression analysis were related to community pharmacists and their workload characteristics. Future studies may include specific patient-related factors to assess if these are also important determinants of community pharmacists’ minor ailment-related information gathering and counseling practices.

### 4.1 Practice implications

The results of the current study have provided important insights into the significant determinants of community pharmacists’ information gathering and counseling practices during the management of minor ailments, and this may prove useful in identifying appropriate interventions that should be deployed to improve these practices. For instance, assigning more prominent role to female community pharmacists who seem to be better at gathering patient information more comprehensively in any proposed intervention may prove useful. Similarly, the use of community pharmacists in the relatively younger age group to champion interventions targeted to improve counseling practices related to the management of minor ailments seems reasonable and may enhance service delivery.

The observed inconsistency and inadequacy in information gathering and counseling by community pharmacists during the management of minor ailments could potentially compromise patient safety and increase the risks of medication use-related harms. Hence, interventions focused on significant improvement in these critical areas are warranted and will enhance effective and safe management of minor ailments and patient outcomes. For instance, the use of a minor ailment-specific standard protocol may improve community pharmacists’ information gathering and counseling practices and enhance the achievement of positive outcomes.

## 5 Conclusion

The significant determinants of community pharmacists’ Information gathering and counseling practices during the management of minor ailments were Female gender and consultation time (6-10 min), and age group (31–40 years) and consultation time (6–10 min) respectively. Overall, the frequency of Information gathering and provision of counseling information during the management of minor ailments appear inconsistent and requires significant improvement, especially for certain key components.

## Funding

This work was funded by the Office of Research Support, 10.13039/501100004252Qatar University, Doha, Qatar [Grant number: QUST‐1‐CPH‐2021‐13] and Qatar National Research Fund [UREP24-147-3043]. The funding played no role in the design of the study and collection, analysis, and interpretation of data or manuscript writing.

## Declaration of Competing Interest

The authors declare that they have no known competing financial interests or personal relationships that could have appeared to influence the work reported in this paper.
